# Editorial: Neural Computation in Embodied Closed-Loop Systems for the Generation of Complex Behavior: From Biology to Technology

**DOI:** 10.3389/fnbot.2018.00053

**Published:** 2018-08-30

**Authors:** Poramate Manoonpong, Christian Tetzlaff

**Affiliations:** ^1^Embodied AI and Neurorobotics Lab, Centre for BioRobotics, The Mrsk Mc-Kinney Mller Institute, University of Southern Denmark, Odense, Denmark; ^2^Bio-inspired Robotics and Neural Engineering Lab, School of Information Science and Technology, Vidyasirimedhi Institute of Science and Technology, Rayong, Thailand; ^3^Institute of Bio-Inspired Structure and Surface Engineering, Nanjing University of Aeronautics and Astronautics, Nanjing, China; ^4^Bernstein Center for Computational Neuroscience, Third Institute of Physics, Georg-August-Universität Göttingen, Göttingen, Germany

**Keywords:** embodiment, neural computation, sensorimotor coordination, orthosis and prosthesis, motor planning/control, learning and memory, autonomous robot, neuromorphic computing

## 1. Introduction

The brain of biological organisms is a highly complex and very efficient computing unit. It can deal with a multitude of tasks from low-level sensorimotor coordination to high-level cognition. Specifically, it can process high-dimensional sensory information and, dependent on this, generate coordinated motor commands in real time, resulting in actions (like, locomotion and manipulation). Simultaneously, it can also perform cognitive functions (such as navigation, goal-oriented behavior, reasoning and decision making, interaction, communication). This amazing performance is achieved by using the full capacity of its neural dynamics, learning, memory, and adaptation as well as by interacting with the environment through its body (i.e., sensory-motor system). Thus, actions and cognition require dynamical brain-body-environment interactions and thereby cannot be disembodied. A traditional view of embodiment has also emphasized that complex behavior emerges from continuous and dynamical interactions between computational and physical means with the environment (Wilson, 2002; see also the embodiment scheme in Pfeifer et al., 2007). While this radical scientific concept has been promoted since the last three decades (Brooks, 1991; Chiel and Beer, 1997; Calvo and Gomila, 2008; Pfeifer et al., 2014), the detailed interaction of the (neural) computation within and across different brain areas, as the sensory, motor, and higher integrative areas, with the environment to generalize complex and adaptive behaviors have not been fully addressed.

**Figure 1:**
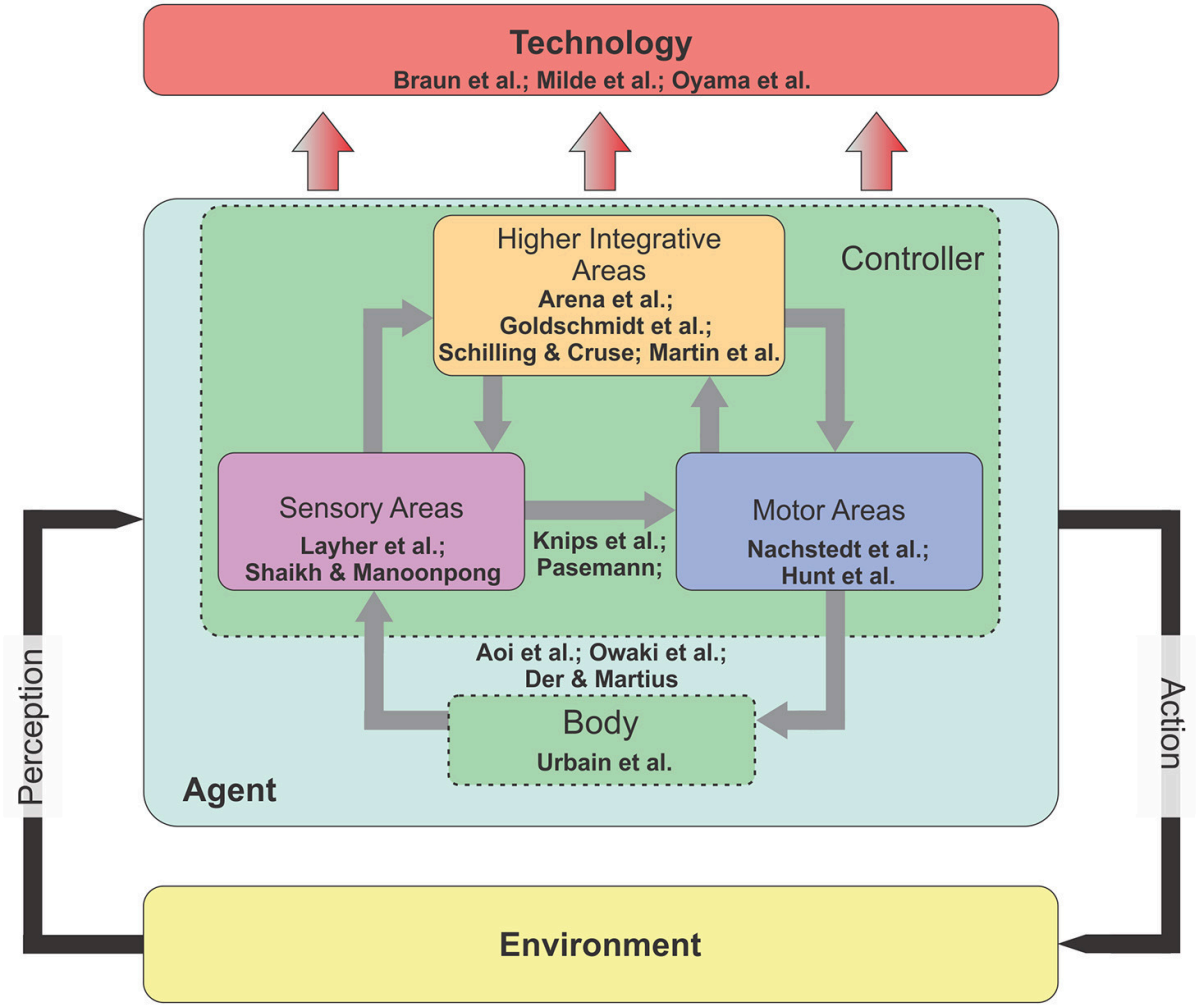
The diagram of an embodied closed-loop system. The system concerns an agent that is situated in the environment. It can perceive the environmental information through its sensors and perform its actions using its motors. In principle, the agent consists of two main components: Nervous system (or controller) and body. There are three main areas inside the nervous system: Sensory, motor, and higher integrative areas. In the embodied perspective, while all external and internal stimuli are processed in the nervous system, this computational processing can be offloaded to the body (i.e., morphological computation) for a successful interaction with the environment (see text for more details).

According to this, this Research Topic called researchers from different fields (including Biology, Computational Neuroscience, Robotics, and Artificial Intelligence) to share their recent developments and results and to update our research community with remaining open questions. The topic has in total 17 articles which cover neural and morphological computations as well as the transfer of results to real world applications, like prosthesis and orthosis control and neuromorphic hardware implementation. Eight articles focus on the three main areas (sensory, motor, and integrative areas) of the controller or brain (Figure 1). Among these, two focus on neural computation mechanisms in sensory areas for action recognition of a human agent

(Layher et al.) and for acoustic motion perception (Shaikh and Manoonpong), twointegrative areas for motor-skill learning (Arena et al.), navigation learning (Goldschmidt et al.), motor planning and internal representations (Schilling and Cruse), and self-organized complex locomotion patterns (Martin et al.). In addition, two articles present neural closed-loop architectures that link between sensory and motor areas for reaching and grasping (Knips et al.) and for, e.g., obstacle avoidance behavior (Pasemann). Three articles consider a tight interaction between the body and the sensory and motor areas for sensorimotor coordination of legged robots (Aoi et al.; Owaki et al.) and a robot arm (Der and Martius). One article provides an insight on the computation of morphological body for optimal locomotion learning (Urbain et al.). Regarding to technology transfer, two articles show the transfer of the principles of the nervous system for orthosis (Braun et al.) and prosthesis control (Oyama et al.) and one shows the transfer to neuromorphic hardware-based control (Milde et al.). Based on these contributions, we organize subsections into two main categories: Embodied closed-loop systems and their technology transfer.

## 2. Overview

### 2.1. Embodied closed-loop systems

An embodied closed-loop system or a brain-body-environment system (Chiel and Beer, 1997) is generally formed by three main ingredients: Nervous system (or controller), body, and the environment. The nervous system has in general three subareas: Sensory, motor, and higher integrative areas. Environmental information is perceived through sensors and processed in the sensory areas. The sensory areas can be also influenced by forward internal models (Kawato, 1999) embedded in the higher integrative areas for sensory prediction and noise cancellation (von Holst and Mittelstaedt, 1950; Blakemore et al., 1999) as well as state estimations (Frens and Donchin). The outputs of the sensory areas are transmitted to motor and higher integrative areas. The motor areas translate the sensory information into motor commands to control the body. They also send a copy of motor commands (efference copy) to the forward models. The higher integrative areas have multiple complex functions with multiple-time scales plasticity (i.e., long-term and short-term synaptic plasticity) for motor-skill learning, navigation learning, motor planning, and internal representations. Their outputs project to both sensory and motor areas. Through these internal (inside the controller) and external (between the body and the environment) interactions in the closed-loop system, adaptive behaviors emerge.

Regarding interactions in embodied closed-loop systems for adaptive behavior generation, in the Research Topic, Aoi et al. review various adaptive locomotor behaviors that emerge from the interactions between the body dynamics, the nervous system, and the environment in animals and legged robots. They identify key factors and mechanisms for adaptations to different speeds, environmental situations, body properties, and tasks. The factors and mechanisms include CPGs, sensory feedbacks, forward model, learning model, and muscle stiffness. Owaki et al. present a novel and minimal Tegotae-based approach, that exploits the interactions between foot contact sensor signals, body dynamics, and the environment for adaptive interlimb coordination of a hexapod robot. The approach can generate various insects' gait patterns that allows the robot to adapt to different locomotion speeds, changes in the weight distribution, and leg amputation. Der and Martius report self-organized behavior of an anthropomorphic musculoskeletal robot arm. The behavior emerges from the interaction between a self-learning neural controller (nervous system), the elastically musculoskeletal arm (body), and an object (environment) through proprioceptive sensory feedback. Through the agent-environment coupling, the robot can perform handshaking, pendulum swinging, bottle shaking, rotating a wheel, wiping a table, and hand-eye coordination.

As a part of embodied closed-loop systems, dynamical system-based architectures that link between sensory and motor areas are introduced in the Research Topic. Knips et al. present a neural dynamic architecture for reaching and grasping objects. It integrates several modules, having functions for scene representation, concurrent object classification and pose estimation, behavioral organization, and movement generation, into a large dynamical system of an anthropomorphic robot arm equipped with a Kinect sensor. In addition to the perception, integration, and movement generation, the architecture can also allow for the online adaptation of the performed movement of the robot for successful completion of the grasp. Pasemann proposes the exploitation of the discrete-time neurodynamics of networks in a sensorimotor loop for generating adaptive behavior, like adaptive obstacle avoidance of mobile robots. The behavior generation is a result from a projection of attractor transients or meta-transients, embedded in neurodynamics, to the motor space.

#### 2.1.1. Sensory areas

In a closed-loop scenario, agents have to extract and process relevant information from the environment, they are situated in. For this, the initial step is to perceive at least parts of the environment via the sensory system. Thereby, the sensory modality can be multifaceted and requires various types of sensors in the system, as for visual, touch, or sound perception. As the next step, the sensory system has to preprocess the perceived environmental information to support the computation done in subsequent areas. Based on experimental insights from the lizard auditory system (Fletcher and Thwaites, 1979; Christensen-Dalsgaard and Manley, 2005), Shaikh and Manoonpong developed a model of the auditory system that can learn to perceive a sound, and to process the information to localize its source and to estimate the speed and direction of the motion of the source. Different to previous approaches, the model can track the source, although it is occluded for a certain duration. By using the model for the auditory system of a wheeled robot, the robot was always able to perceive, to locate, and, by its sensor-motor interaction, to face the sound source.

The extraction of more abstract but complex concepts from the environmental stimulus stream requires, in general, a larger and more sophisticated sensory system. Layher et al. trained a deep neural network to recognize human poses from a stream of images. The pose recognition is based only on features of human body motions and shapes not requiring feedback from higher integrative areas. By implementing the network on a neuromorphic hardware, the recognition process becomes real time with about 1,000 frames per second. Remarkably, the system already shows indications of generalization of poses.

#### 2.1.2. Motor areas

Central pattern generators (CPGs) have been identified as one of key mechanisms in the motor areas particulary for locomotion control. The principle of biological CPGs has been widely used for robot locomotion control (Ijspeert, 2008). Although CPGs do not need any external input or feedback to produce basic rhythmic activity, they still require sensory feedback to adapt and tune their produced activity, e.g., their frequency or phase. Reactive and adaptive mechanisms have been introduced for this purpose (Buchli et al., 2006). A reactive mechanism can entrain the CPG signal where the frequency of the signal matches to sensory feedback. However, if the feedback disappears, the CPG signal will return to its intrinsic frequency. By contrast, an adaptive mechanism modifies the intrinsic frequency of the CPG permanently. Here, Nachstedt et al. propose a novel frequency adaptation mechanism through fast dynamical coupling (AFDC) of a CPG model. It is an extension of the standard frequency adaptation mechanism (Righetti et al., 2009) and based on dynamically adapting the coupling strength of sensory feedback to a CPG model. Using this AFDC technique, they achieve fast and precise adaptation for a wide range of initial intrinsic and target frequencies without the need for parameter fine tuning.

Hunt et al. report a CPG-based motor control circuit with sensory feedback and an automatic process for neural parameter setting. It is based on the known connectivity of mammalian locomotor systems. The process, faster and more reliable than manual tuning, can tune neural parameters to generate adaptive locomotion in the rear legs of a dog-like robot driven by artificial muscles. Using the CPG-based control approach, they show that the dog-like robot can adapt its stepping continuously and maintains rhythmic walking.

#### 2.1.3. Higher integrative areas

Different to sensory and motor areas, higher integrative areas are associated with cognitive processes as learning, planning, navigation, or generalization. For instance, Goldschmidt et al. developed a system, which learned in a reward-based manner to represent the path an agent has walked. By this, the agent is able to robustly localize itself within the environment and to find back to the home position. Thereby, the resulting behaviors are quite similar to behaviors of insects as desert ants.

Interestingly, the neuronal network underlying cognitive processes can be quite small by using the computational resources of other areas or by utilizing the temporal dynamics of the system (Buonomano and Maass, 2009). Martin et al. use the ongoing dynamics of short-term synaptic plasticity (Tsodyks and Markram, 1997) in a three-neuron system to switch between different, complex motor patterns. Here, Schilling and Cruse show that already a small neuronal network is sufficient for successful planning within an environment and generalization to other environments, if the system recruits and orders the resources of the downstream motor areas. In other words, the small network reorders diverse reactive behaviors, each stored in a different part of the motor area, to adapt according to new environments.

There is a clear indication that higher integrative areas are not mandatory for cognitive processes (Cruse and Wehner, 2011). Arena et al. show in a theoretical model that learning within the Drosophila mushroom body, which is in general associated with the sensory processing of olfactory inputs, adapts motor commands or primitives in the motor area. Thus, by changes in the sensory area, the sensory-motor relations are updated yielding new behaviors. This was demonstrated on a six-legged robot, which can learn by this mechanism to climb up stairs. The authors also address the role of Neural Reuse as one of the possible keys for the emergence of complex behaviors in simple brains.

#### 2.1.4. Body

Apart from neural computation in the nervous system, morphological computation also contributes to the generation of complex behavior. Morphological computation considers that certain processes can be performed by the body instead of the nervous system (Pfeifer and Bongard, 2006). In other words, it captures conceptually the observation that biological systems utilize their flexible and compliant morphology to conduct computations required for a successful interaction with their complex environments. There are numerous illustrative applications of morphological computation and embodiment for efficient locomotion in biological systems (Dickinson et al., 2000) and artificial systems (McGeer, 1990; Jayaram and Full, 2016; Manoonpong et al., 2016). Here, Urbain et al. present an analysis of the trade-offs between morphology, efficiency of locomotion, and the ability of a mechanical body. This is done by using a detailed dynamical model of a Mass-Spring-Damper (MSD) network. They also analyze the computational capacity of a MSD body to generate motor control signals and integrate the signals as feedback to a locomotion controller.

### 2.2. Technology transfer

Analyzing the neural computation in closed-loop systems, on the one hand, yields insights of the underlying neural dynamics and principles and, on the other hand, provides new solutions for technological control problems. Braun et al. developed a neural controller which tracks and predicts the gait of a patient to control the gait-supporting knee-ankle-foot orthosis. This controller is independent of the actual environmental situation, as walking on a flat terrain or climbing stairs, and requires a minimal feedback from the patient.

Based on adaptive principles in neural circuits, Oyama et al. developed an adaptive controller for a hand prosthesis. A standard controller requires the user to interfere to avoid errors given in different environmental conditions. By contrast, the adaptive controller self-adapts according to the new environmental state or different hand poses.

Milde et al. transferred the neural controller and the whole neural sensory processing onto neuromorphic hardware. By implementing this hardware architecture, they developed an autonomous, neuromorphic robotic agent, which is able to avoid obstacles and to acquire targets. Due to the neural nature of the controller, the agent behaves robustly according to unexpected changes in the environment.

## 3. Conclusion

The Research Topic presents an embodied closed-loop approach that considers the interaction of (neural) computation across sensory, motor, and higher integrative areas with the agent's body and the environment. The studies in this Topic cover the broad spectrum of this approach and show that, indeed, complex behaviors emerge from the interplay between different parts of an agent. Thereby, the majority of these studies focus on the interplay between a subset of the available parts. The results from these studies confirm that the embodied approach can be a powerful method to develop autonomous robotic agents performing complex behaviors and it can even be a key to understand high-level cognition. Given these and further studies, it is now possible to address the interaction between all parts of an agent's controller (brain), body, and the environment.

## Author contributions

All authors listed have made a substantial, direct and intellectual contribution to the work, and approved it for publication.

### Conflict of interest statement

The authors declare that the research was conducted in the absence of any commercial or financial relationships that could be construed as a potential conflict of interest.
